# Multivariate Analysis of Effects of Asthmatic Patient Respiratory Profiles on the *In Vitro* Performance of a Reservoir Multidose and a Capsule-Based Dry Powder Inhaler

**DOI:** 10.1007/s11095-015-1820-1

**Published:** 2015-11-16

**Authors:** Francesca Buttini, Irene Pasquali, Gaetano Brambilla, Diego Copelli, Massimiliano Dagli Alberi, Anna Giulia Balducci, Ruggero Bettini, Viviana Sisti

**Affiliations:** Department of Pharmacy, University of Parma, Viale delle Scienze 27/A, 43124 Parma, Italy; Institute of Pharmaceutical Science, King’s College London, 150 Stamford Street, SE19NH London, UK; Chiesi Farmaceutici SpA, Via Palermo 26, 43122 Parma, Italy; Interdepartmental Center, Biopharmanet-TEC, University of Parma, Viale delle Scienze 27/A, 43124 Parma, Italy

**Keywords:** Multivariate analysis, Breath simulator, Alberta throat, NEXThaler^®^, RS01

## Abstract

**Purpose:**

The aim of this work was to evaluate the effect of two different dry powder inhalers, of the NGI induction port and Alberta throat and of the actual inspiratory profiles of asthmatic patients on *in-vitro* drug inhalation performances.

**Methods:**

The two devices considered were a *reservoir* multidose and a capsule-based inhaler. The formulation used to test the inhalers was a combination of formoterol fumarate and beclomethasone dipropionate. A breath simulator was used to mimic inhalatory patterns previously determined *in vivo*. A multivariate approach was adopted to estimate the significance of the effect of the investigated variables in the explored domain.

**Results:**

Breath simulator was a useful tool to mimic *in vitro* the *in vivo* inspiratory profiles of asthmatic patients. The type of throat coupled with the impactor did not affect the aerodynamic distribution of the investigated formulation. However, the type of inhaler and inspiratory profiles affected the respirable dose of drugs.

**Conclusions:**

The multivariate statistical approach demonstrated that the multidose inhaler, released efficiently a high fine particle mass independently from the inspiratory profiles adopted. Differently, the single dose capsule inhaler, showed a significant decrease of fine particle mass of both drugs when the device was activated using the minimum inspiratory volume (592 mL).

## Introduction

Breath-actuated dry powder inhalers (DPIs) and metered dose inhalers are the most used systems to deliver locally-acting drugs to the lungs. DPIs are becoming more and more popular because they do not need the coordination between release of the dose and patient inhalation manoeuvre, they do not contain any propellant and dry powders are generally more stable than liquid formulations (1–3). Since their market introduction, many different devices have been developed in order to obtain systems with: i) a performance largely independent of the air flow rate through the device; ii) good de-aggregation properties at achievable patient breath rate; iii) reliable and consistent dose delivery (4–6).

Notwithstanding the improvements introduced in the last decades, the amount of drug administered by several DPI still depends on the inhalation profile of the patient. The European Guideline on the quality of pharmaceutical products for inhalation clearly states that to assess the correct performance of a DPI, both the uniformity of delivered dose and the respirable mass (particles below 5 μm) must be determined in the range of inspiratory flow rates achievable by the patients for whom the product could be prescribed and that this range must be justified in relation to the clinical trials (7). To address this point, it is suggested that consideration should be given to at least three inspiratory flow (e.g. the tenth, fiftieth and ninetieth percentile of the inspiratory profiles of a population of patients) (8).

In this respect, recently the inhalation profiles from asthmatic patients through the NEXThaler device determined by means of a validated acoustic monitoring equipment (Sensohaler^®^ Sagentia Inc. Cambridge, UK) (9), have been published. In this study, the 10^th^, 50^th^ and 90^th^ percentile cohort values were calculated at each time point interval (0.01 s) and the relevant flow curves generated. The two individual profiles exhibiting the lowest and highest peak inspiratory flow (PIF) values were also produced (10).

On the other hand, the flow profile used during the aerodynamic size assessing according to the European Pharmacopoeia or the USP is based on the capability of the equipment to get instantaneously to a set flow, dependent on the resistance of the inhaler, then to keep it constant for the time necessary to inhale a volume of up to 4 L. This method, while very useful for a QC characterization of DPIs, is clearly not representative of what happens *in vivo*.

The attempt to obtain *in vitro* results more representative of *in vivo* conditions, led to the recent development of breath simulators to test orally inhaled and nasal drug products. One of such breath simulator, namely a pump equipped with a microprocessor instrument able to generate inspiratory profiles similar to those of patients with a maximum inspired volume of 5 L, is available on the market (8) and was selected for experimental plan of the present work.

Furthermore, the evaluation of the extra-fine particle mass (<2 μm) was suggested as a tool for better *in vitro*-*in vivo* correlation (11,12).

Another aspect of the standard aerodynamic size analysis that has recently been deeply investigated is the geometry of the Ph. Eur./USP common induction port (IP), used to connect inhalers to the cascade impactors (13). Recently the Alberta throat (AT), an idealized connectors developed on the basis of anatomical data of typical patient population (14–16) has been proposed with the aim to provide a more realistic representation of the human throat in the *in vitro* aerodynamic assessment. All experiments of the present work were performed with both IP and AT to evaluate the effect of these two different types of connections on the aerodynamic drug performance.

Finally, the inhalation device has the important role to deliver and generate aerosol clouds containing particles able to penetrate in the respiratory tract. There are many different types of dry powder inhalers that can be differentiated on the base of how they store the formulation, i.e.: multi dose reservoir devices that can meter the dose when the device is actuated or capsule driven ones where the single doses are pre-metered in hard capsules. (5,6,17).

The guideline ICH Q8 “Pharmaceutical Development” recommends the use of systematic methods based on risk analysis and management, to identify the variables that can affect the critical quality attributes of new medicine (18). Therefore, for the development of a new medicinal product an approach based on the use of experimental designs and techniques for the identification, assessment and management of risks is required. The experimental design is used to evaluate the effect of the variables and their interactions on specific outcomes. It allows planning the experiments in order to obtain the maximum information with the minimum number of experiments.

The hypothesis behind the present work was the possible inter-correlation among the above cited factor affecting the *in vitro* aerodynamic behaviour of an inhalation powder when aerosolized with two different devices.

The aim of this work was to evaluate, by means of a multivariate approach, the effect of two different dry powder inhalers, of the common induction port and Alberta throat and of the actual inspiratory profiles of asthmatic patients (generated with a breath simulator) on *in-vitro* inhalation performances. The two devices considered were a multidose inhaler and a single dose capsule inhaler having a similar resistance. The formulation used to test the inhalers was a combination of a β_2_-agonist molecule (formoterol fumarate, FF) and a topical anti-inflammatory agent (beclomethasone dipropionate, BDP).

## Materials and Methods

### Materials

A combined dry powder formulation containing formoterol fumarate (FF) and beclomethasone dipropionate (BDP), blended with alpha lactose monohydrate as a carrier (212-255 μm), was employed in the study. The ratio among the components was such that for 10 mg dose of powder 6 μg of FF and 100 μg of BDP were provided. The used samples were taken from standard production batches (Chiesi Farmaceutici S.p.A., Parma, Italy). The formulation was either introduced in a multidose device (NEXThaler^®^, Chiesi Farmaceutici S.p.A., Parma, Italy) or used to fill the capsules of a pre-metered inhaler (RS01, Plastiape, Lecco, Italy). The tested devices had a similar internal flow resistance ranging between 0.033 and 0.036 √kPa / (L min^−1^). Each capsule (size 3, hypromellose, Capsugel, Colmar, France) was hand filled with 10 mg of dry powder inhalation and each NEXThaler^®^, releasing 10 mg/dose, was filled with 1.5 g of dry powder for inhalation.

FF, supplied by Industriale Chimica Srl (Varese, Italy), and BDP, supplied by Farmabios S.p.a. (Pavia, Italy), were used to prepare standards for analytical quantification by high performance liquid chromatography (HPLC). All solvents used were HPLC grade (Sigma Aldrich, Poole, UK). Ultrapure water was produced by reverse osmosis (MilliQ, Millipore, Molsheim, France).

### FF and BDP assay

All samples were quantified by HPLC as previously described (12, 19). Briefly, the HPLC system (Waters Alliance–Waters Corporation, MA, USA) was equipped with a quaternary pump, auto-sampler, degasser and a variable wavelength UV detector. The analysis were performed using as stationary phase an Atlantis C-18 column, 3 μm, 150 × 3.9 mm (Waters Corporation, MA, USA), adopting the following conditions: flow rate 1.0 mL/min, injection volume 50 μL and column temperature of 40°C. The wavelength was set at 223 nm from 0 to 6.5 min for FF detection and shifted to 238 nm from 6.5 to 12 min for BDP revealing. Mobile phase was prepared by mixing phosphate buffer NaH_2_PO_4_ 0.02 M adjusted to pH of 3.0 and acetonitrile in ratio 75:25 and a gradient program was set to separate and quantify FF and BDP respectively.

### *In vitro* Aerodynamic Performance

#### Cascade Impactor Analysis

The aerodynamic assessment of the dried powders was carried out using the Next Generation Impactor (NGI) (Copley Scientific, Nottingham, UK). The NGI divides the particles discharged from the inhaler into fraction with different particle sizes. The Fine Particle Mass is constituted of the particles having an aerodynamic diameter lower than 5 μm, while particles with an aerodynamic diameter lower than 1 μm were considered in this work representative of the Extra-Fine Particle Mass (EFPM). The Fine Particle Fraction and Extra-Fine Particle Fraction are calculated, as a percentage of Delivered Dose, from the FPM and EFPM values, respectively.

An amount of 10 mg of powder, accurately weighed, was manually introduced into a size 3 HPMC capsule. The capsule was then inserted into the holder chamber of the DPI device and pierced before the performance evaluation. The NEXThaler^®^ was filled with 1.5 g of dry powder in the reservoir chamber. Three capsules and three shots for NEXThaler^®^ were discharged for each NGI assessment. The plates of the impactor were coated with ethanol solution containing 2% w/v of Tween 20 to prevent particle bounce. The drug deposited inside the impactor was collected with a mixture of water:methanol (40:60) and samples analysed by HPLC. NGI experiments were performed in triplicate.

### Breath Simulator

NGI tests were performed using specific inspiratory profiles generated with a breath simulator as well as with the standard procedure required by USP 38 and Ph. Eur. 8. In the latter case, the device was connected to the NGI and passed by the air stream at 60 L/min capable to generate a pressure drop of 4 kPa through the device and activated for 4 s to let 4 L air passing through the system.

**Fig. 1:**
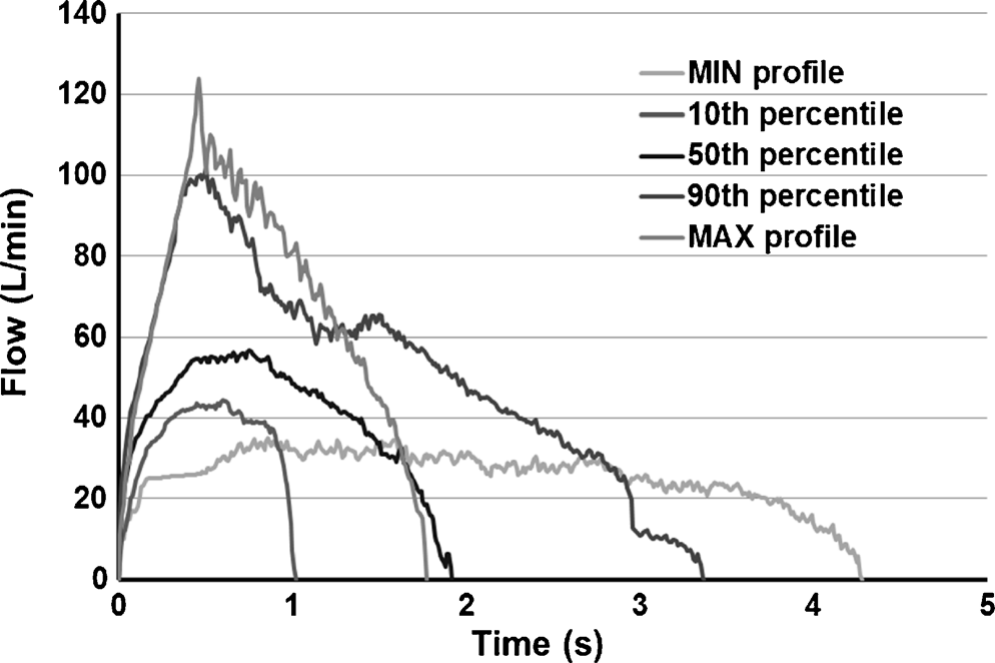
Inspiratory profiles obtained from asthmatic patients inhaling twice through the NEXThaler^®^. Profiles corresponding to the 10^th^, 50^th^ and 90^th^ percentile of the Peak Inspiratory Flow (PIF) as well as the profile with the maximum PIF (MAX profile) and the one with the minimum PIF (MIN profile) are illustrated.

Aerodynamic particle size distribution of FF and BDP was then tested using a breath simulator (BRS 3000, Copley Scientific, Nottingham, UK) able to generate specific inhalation profiles by the use of two pumps that provide a specific breathing pattern to the device while maintaining a constant flow in the impactor. The simulator was set in order to mimic inhalatory patterns previously determined in the clinical trial where 41 asthmatic patients were asked to inhale twice through the NEXThaler^®^ (10). 40 patients were included in the study. 24 of them (60%) were female and 16 (40%) were male. All except one patient (2.5%) were Caucasians. The patients were on average 43 years old (18–77 years range). Their mean body weight was 71.1 kg and the mean body mass index was 25.3 kg/m^2^. Five inspiratory profiles were selected to be reproduced *in vitro*, namely those corresponding to the 10^th^ percentile, 50^th^ percentile and 90^th^ percentile of the Peak Inspiratory Flow (PIF) obtained in the study population, as well as the profile with the maximum PIF (MAX profile) and the one with the minimum PIF (MIN profile) (Fig. 1).

### Design of Experiment

A Design of Experiment approach and Multivariate Data Evaluation by a Principal Component Analysis were used in this study to estimate the significance of the investigated variables in the explored domain. The explored variables and their investigated levels were:

1. Inspiratory profile, at six levels, namely the reference conditions according to Ph. Eur., MIN profile, 10^th^, 50^th^, 90^th^ percentile and MAX profile. Each profile was associated with the corresponding inspiratory peak inhalation, the duration of inhalation and the volume of the inspiration;
2. Type of throat, at two levels, namely IP and “Alberta throat”;
3. Type of inhaler, at two levels, namely capsule inhaler and multidose inhaler.

To study the whole domain identified by the three variables and their levels, 24 sets of experiments (6 × 2 × 2) were required (Table I). Each experiment was performed in triplicate, obtaining an experimental matrix of 72 experiments (Table II). The 72 experiments were carried out according to a randomized order to avoid systematic errors that could alter the results.

**Table I:** Scheme of the Design of Experiment Consisting of Three Variables: Type of Inhaler, Type of Throat and Inspiratory Profile, (IP=Induction Port)

Type of inhaler	Type of throat	Inspiratory profile
NEXThaler^®^	IP	Reference Condition
		MIN profile
		10^th^ percentile
		50^th^ percentile
		90^th^ percentile
		MAX profile
	Alberta throat	Reference Condition
		MIN profile
		10^th^ percentile
		50^th^ percentile
		90^th^ percentile
		MAX profile
Capsule Inhaler	IP	Reference Condition
		MIN profile
		10^th^ percentile
		50^th^ percentile
		90^th^ percentile
		MAX profile
	Alberta throat	Reference Condition
		MIN profile
		10^th^ percentile
		50^th^ percentile
		90^th^ percentile
		MAX profile

**Table II:** Experimental Plan of 24 Different Experiments Performed in Triplicate (IP=Induction Port)

Exp. n.	Inspiration volume (mL) (X_1_)	Type of throat (X_2_)	Type of inhaler (X_3_)
1–3	1838	IP	NEXThaler^®^
4–6	1838	Alberta throat	NEXThaler^®^
7–9	1838	IP	Capsules Inhaler
10–12	1838	Alberta throat	Capsules Inhaler
13–15	592	IP	NEXThaler^®^
16–18	592	Alberta throat	NEXThaler^®^
19–21	592	IP	Capsules Inhaler
22–24	592	Alberta throat	Capsules Inhaler
25–27	1303	IP	NEXThaler^®^
28–30	1303	Alberta throat	NEXThaler^®^
31–33	1303	IP	Capsules Inhaler
34–36	1303	Alberta throat	Capsules Inhaler
37–39	2894	IP	NEXThaler^®^
40–42	2894	Alberta throat	NEXThaler^®^
43–45	2894	IP	Capsules Inhaler
46–48	2894	Alberta throat	Capsules Inhaler
49–51	2050	IP	NEXThaler^®^
52–54	2050	Alberta throat	NEXThaler^®^
55–57	2050	IP	Capsules Inhaler
58–60	2050	Alberta throat	Capsules Inhaler
61–63	4000	IP	NEXThaler^®^
64–66	4000	Alberta throat	NEXThaler^®^
67–69	4000	IP	Capsules Inhaler
70–72	4000	Alberta throat	Capsules Inhaler

The design space was constructed and analysed using JMP^®^ Software, Version 9.0.2 (SAS, Cary, NC, USA). The results were also elaborated using programs written for the Matlab environment (MathWorks, Natick, MA, USA).

### Data analysis

The collected responses of the experimental design were 24 different Critical Quality Attributes (CQAs). Twelve CQAs both for formoterol fumarate and beclomethasone dipropionate were analysed (Table III).

**Table III:** List of Critical Quality Attributes (CQAs) for Formoterol Fumarate and Beclomethasone Dipropionate

CQAs	Reference number
FF Fine Particle Mass	1
FF Extra Fine Particle Mass	2
FF Fine Particle Fraction	3
FF Extra Fine Particle Fraction	4
FF Mass Median Aerodynamic Diameter	5
FF Particle Mass size 0–1 μm	6
FF Particle Mass size 1–2 μm	7
FF Particle Mass size 2–3 μm	8
FF Particle Mass size 3–4 μm	9
FF Particle Mass size 4–5 μm	10
FF throat deposition	11
FF Delivered Dose	12
BDP Fine Particle Mass	13
BDP Extra Fine Particle Mass	14
BDP Fine Particle Fraction	15
BDP Extra Fine Particle Fraction	16
BDP Mass Median Aerodynamic Diameter	17
BDP Particle Mass size 0–1 μm	18
BDP Particle Mass size 1–2 μm	19
BDP Particle Mass size 2–3 μm	20
BDP Particle Mass size 3–4 μm	21
BDP Particle Mass size 4–5 μm	22
BDP throat deposition	23
BDP Delivered Dose	24

Fine Particle Mass, Extra Fine Particle Mass, Fine Particle Fraction, Extra Fine Particle Fraction and Mass Median Aerodynamic Diameter were measured or calculated according to Ph. Eur. 8 ed. specifications. The throat deposition was the amount of active ingredients recovered in the throat (IP or Alberta throat). The particle masses in selected size ranges were characterized by different particle size and calculated by CITDAS Copley Scientific software.

## Results

### Critical Quality Attributes

Twenty-four Critical Quality Attributes (CQAs) of the formulation produced were selected.

The fine particle mass is the amount of drug particles smaller than 5 μm deposited in the impactor. It is the most significant attribute to consider and it indicates the product respirability. The delivered dose is another important critical quality attribute to take into consideration because it is related to the drug dose leaving the device upon inhalation. The Ph. Eur. specification establishes that more than 75% of the loaded dose must leave the device upon aerosolization in compendial conditions (20). The powder size, expressed as volume diameter of particles, reflects the capability of the powder production to provide a particle size distribution at micron level, in order to obtain a favourable aerodynamic diameter. In fact, the volume diameter is the most relevant parameter determining the aerodynamic size of particles, which is the critical spherical equivalent diameter to refer to inhalation formulation.

The results were evaluated with multivariate analysis. The Principal Components Analysis (PCA) allows extracting the information contained in the data showing correlations between experimental variables, possible trends and potential outliers. PCA is characterized by two main outputs: loading plot and score plot.

**Fig. 2:**
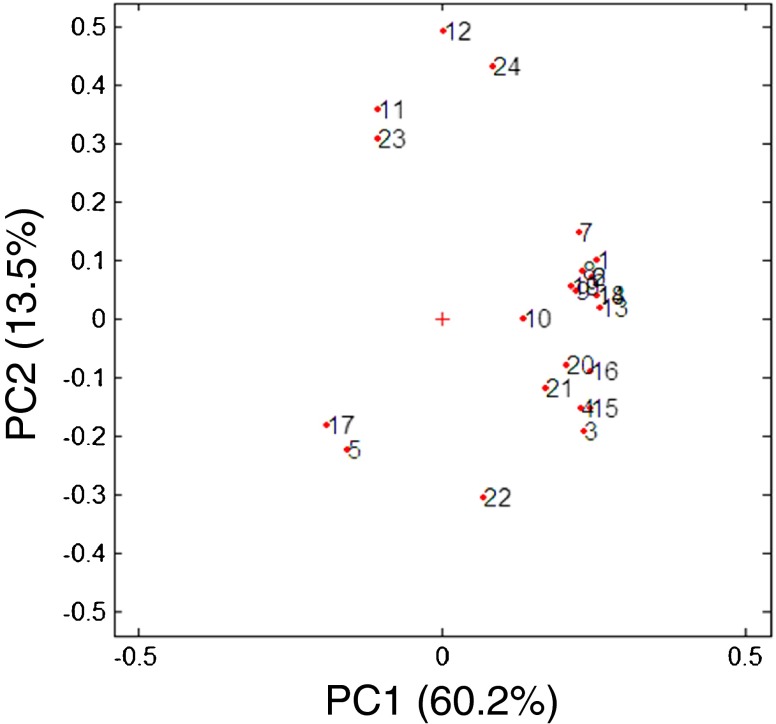
Loadings plot representing the PC1 and PC2, collecting 60.2% and 13.5% of the variance, respectively.

The loading plot (Fig. 2) represents the projection of the original variables in the subspace dimension. The coefficients (loadings) of the linear combination correspond to each principal component.

The loadings define the orientation of PC plane with respect to the original variables. They explain how the variables are linearly combined to form the scores. The loadings unravel the magnitude (big or small) and the way (positive or negative correlation) in which the variables contribute to the scores.

The scores are the coordinates of the linear combinations, therefore the score plot allows to visualize the position of each observation in the subspace of the principal components. In the loading plot of Fig. 2 the *x*-axis representing the PC1, collects most of the variance (60.2%) compared to PC2 (13.5%) on the *y*-axis. Since PC1 collects about 4 times more variance than PC2, equal distance on PC1 is 4 times more important than on PC2.

The PC1 explains the results related to the aerodynamic particles distribution (for FF points 1–10, for BDP 13–22), while PC2 explains the variables throat deposition (11 for FF and 23 for BDP) and Delivered Dose (12 for FF and 24 for BDP). These four variables (11, 23, 12 and 24) are related among each other, i.e. high value of delivered dose led to high deposit on the throat surface due the consistent amount of powder released. All the results of FF are related to BDP ones. For example, the position of the FF MMAD (5) is very close to that of the BDP (17). This means that the two active ingredients have equivalent behaviour and could be potentially co-deposited in the respiratory tract of patients. Among the collected responses, eighteen of them, namely FPM, EFPM, FPF, EFPF, Particle size 0–1 μm, Particle size 1–2 μm, Particle size 2–3 μm, Particle size 3–4 μm, Particle size 4–5 μm (1–4 and 6–10 for FF, 13–16 and 18–22 for BDP) are strongly related to each other and are located at positive values of PC1 and therefore are inversely related to the results MMAD of FF and BDP (5 and 17) which are in negative values on the same component. This result implies that if the first group of values increases, the MMAD decreased.

**Fig. 3:**
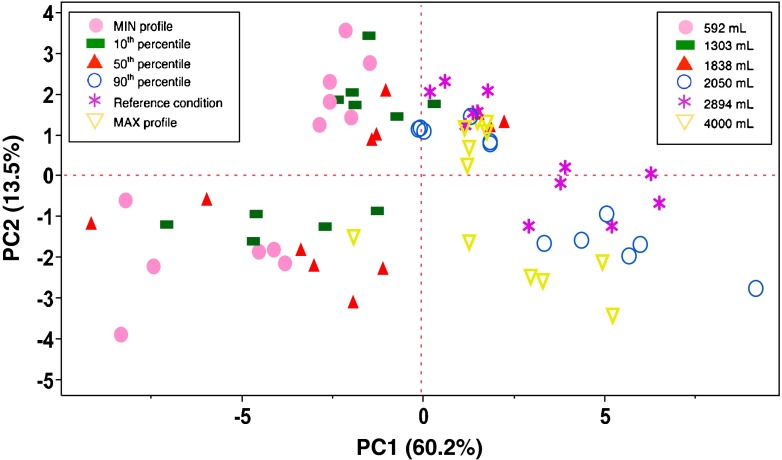
Scores plot: the effect of inspiratory profiles and inspiration volume on aerodynamic performances.

The Score plot of Fig. 3 underlines the effect of the five inspiratory profiles as well as of the reference Pharmacopoeia profile. Two families of data are explained on PC1: the first, at low values on PC1, which collects the experiments performed with the MIN profile, 10^th^ percentile and 50^th^ percentile (these experiments have high values of the MMAD and low values of FPM, EFPM, FPF, EFPF, Particle size 0–1 μm, Particle size 1–2 μm, Particle size 2–3 μm, Particle size 3–4 μm, Particle size 4–5 μm) and a second family, with high values on PC1, which collects experiments performed with the inspiratory profiles 90^th^ percentile, MAX profile and the reference condition (these experiments have low values the MMAD and high values of, FPM, EFPM, FPF, EFPF, Particle size 0–1 μm, Particle size 1–2 μm, Particle size 2–3 μm, Particle size 3–4 μm, Particle size 4–5 μm). This data evidenced that independently on the type of throat or inhalation device, the *in vitro* inhalation performance of 90th percentile and MAX profile are equivalent to that of reference condition, while the MIN profile, 10th percentile and 50th percentile showed MMAD higher than the reference condition whereas the FPM, EFPM, FPF, EFPF, Particle size 0–1 μm, Particle size 1–2 μm, Particle size 2–3 μm, Particle size 3–4 μm, Particle size 4–5 μm were lower than the reference condition.

Each inspiratory profile as well as the reference condition can be described by the peak of inhalation, duration and volume of inspiration, thus the Scores plot of Fig. 3 can be used also to highlight the effect of this latter variable that can be calculated as the area under the curve of inspiration profiles reported in Fig. 1.

In general, regardless of the type of throat or inhaler, the *in vitro* profiles with volumes of inspiration of 2050 and 2894 mL were equivalent to the inhalation performance obtained with the reference condition (4000 mL), while the profiles with total volume of inhalation of 1838, 1303, and 592 mL show MMAD higher than the reference condition and values of FPM, EFPM, FPF, EFPF, Particle size 0–1 μm, Particle size 1–2 μm, Particle size 2–3 μm, Particle size 3–4 μm, Particle size 4–5 μm lower than the reference condition.

**Fig. 4:**
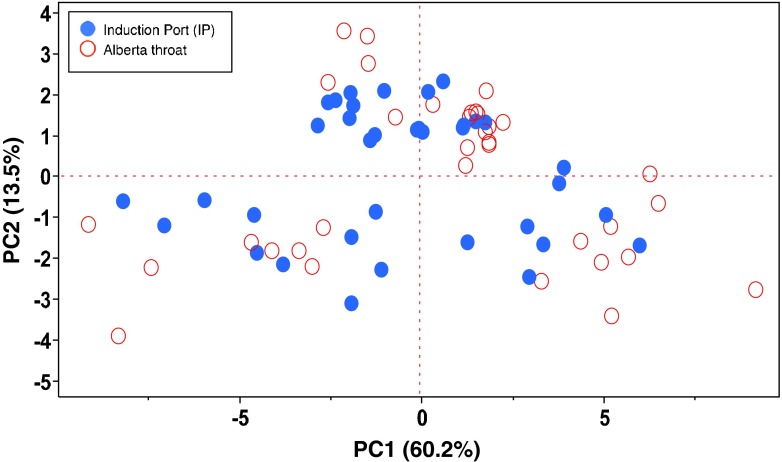
Scores plot: the effect of the type of throat on aerodynamic performances.

In the Scores plot of Fig. 4, relevant to the IP and the “Alberta throat”, all the experiments are mixed randomly within the plot showing that the type of throat used had no impact on the outcomes. In this case it is not possible to identify families of any of the two components.

**Fig. 5:**
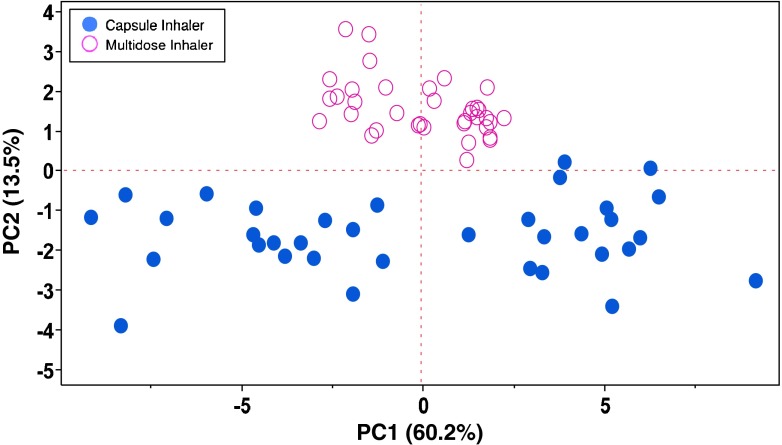
Scores plot: the effect of the type of inhaler on aerodynamic performances.

Finally, it was evaluated the Scores plot of the two types of inhaler (Fig. 5). Contrary to the previous cases, two distinct families of data on PC2 are clearly evidenced: the first, at low values on PC2, which collects the experiments performed with the capsules inhaler (these experiments led to low values of Delivered Dose and amount of drug deposited on IP or “Alberta throat”) and a second family, with high values on PC2, which collects the experiments performed with the multidose inhaler (these experiments led to high values of Delivered dose and amount of drug deposited on IP or “Alberta throat”). This means that in general the multidose allows delivering to the patient a greater amount of drug.

The experiments performed with the capsules inhaler are more distributed along the PC1, thus highlighting a greater variability of the experiments with the capsules inhaler, whereas those performed with the NEXThaler^®^ are less spread highlighting high consistency. Since the PC1 is the component along which the differences between the inspiratory profiles are explained, the observed data mean that the difference in performance between the *in vitro* profiles are much less evident using the multidose inhaler compared the capsule inhaler.

### 3.3. Variable correlation and prediction of the system behaviour

In order to establish a relationship between model matrix X (Table IV) and response y, MLR analysis was carried out. The MLR model is defined by the equation:

1 $$ y = Xb + e $$

where **X** is the model matrix; **b** the coefficient of the model and **e** the error vector. The coefficients of the model can be computed by the following relationship:

**Table IV:** Model Matrix and Responses on PC1 and PC2

Exp. n.	Inspiration volume (mL) (X_1_)	Type of throat (X_2_)	Type of inhaler (X_3_)	PC1	PC2
1	−0.27	−1.00	−1.00	−2.82	1.24
2	−0.27	−1.00	−1.00	−2.54	1.82
3	−0.27	−1.00	−1.00	−1.92	1.43
4	−0.27	1.00	−1.00	−2.09	3.55
5	−0.27	1.00	−1.00	−1.42	2.76
6	−0.27	1.00	−1.00	−2.53	2.29
7	−0.27	−1.00	1.00	−4.46	−1.87
8	−0.27	−1.00	1.00	−8.15	−0.61
9	−0.27	−1.00	1.00	−3.76	−2.15
10	−0.27	1.00	1.00	−7.37	−2.23
11	−0.27	1.00	1.00	−4.06	−1.81
12	−0.27	1.00	1.00	−8.26	−3.90
13	−1.00	−1.00	−1.00	−1.91	2.03
14	−1.00	−1.00	−1.00	−1.84	1.74
15	−1.00	−1.00	−1.00	−2.31	1.87
16	−1.00	1.00	−1.00	0.36	1.76
17	−1.00	1.00	−1.00	−1.44	3.44
18	−1.00	1.00	−1.00	−0.68	1.45
19	−1.00	−1.00	1.00	−1.22	−0.86
20	−1.00	−1.00	1.00	−4.55	−0.95
21	−1.00	−1.00	1.00	−7.01	−1.20
22	−1.00	1.00	1.00	−2.65	−1.24
23^a^	-	-	-	-	-
24	−1.00	1.00	1.00	−4.64	−1.61
25	−0.58	−1.00	−1.00	−1.24	1.01
26	−0.58	−1.00	−1.00	−0.97	2.09
27	−0.58	−1.00	−1.00	−1.38	0.88
28	−0.58	1.00	−1.00	1.90	1.21
29	−0.58	1.00	−1.00	1.59	1.53
30	−0.58	1.00	−1.00	2.27	1.33
31	−0.58	−1.00	1.00	−5.92	−0.57
32	−0.58	−1.00	1.00	−1.87	−3.10
33	−0.58	−1.00	1.00	−1.05	−2.27
34	−0.58	1.00	1.00	−2.97	−2.20
35	−0.58	1.00	1.00	−9.09	−1.18
36	−0.58	1.00	1.00	−3.33	−1.81
37	0.35	−1.00	−1.00	−0.09	1.13
38	0.35	−1.00	−1.00	0.08	1.10
39	0.35	−1.00	−1.00	−0.04	1.17
40	0.35	1.00	−1.00	1.89	0.83
41	0.35	1.00	−1.00	1.88	0.79
42	0.35	1.00	−1.00	1.35	1.46
43	0.35	−1.00	1.00	6.02	−1.69
44	0.35	−1.00	1.00	3.37	−1.66
45	0.35	−1.00	1.00	5.10	−0.93
46	0.35	1.00	1.00	5.71	−1.96
47	0.35	1.00	1.00	9.21	−2.77
48	0.35	1.00	1.00	4.40	−1.57
49	−0.14	−1.00	−1.00	1.79	1.31
50	−0.14	−1.00	−1.00	1.54	1.35
51	−0.14	−1.00	−1.00	1.17	1.20
52	−0.14	1.00	−1.00	1.79	1.09
53	−0.14	1.00	−1.00	1.31	0.70
54	−0.14	1.00	−1.00	1.26	0.27
55	−0.14	−1.00	1.00	−1.88	−1.48
56	−0.14	−1.00	1.00	1.30	−1.60
57	−0.14	−1.00	1.00	2.99	−2.46
58	−0.14	1.00	1.00	4.98	−2.11
59	−0.14	1.00	1.00	5.25	−3.40
60	−0.14	1.00	1.00	3.34	−2.55
61	1.00	−1.00	−1.00	0.62	2.32
62	1.00	−1.00	−1.00	1.20	1.26
63	1.00	−1.00	−1.00	0.23	2.07
64	1.00	1.00	−1.00	1.53	1.59
65	1.00	1.00	−1.00	1.82	2.11
66	1.00	1.00	−1.00	1.41	1.55
67	1.00	−1.00	1.00	3.93	0.21
68	1.00	−1.00	1.00	3.82	−0.18
69	1.00	−1.00	1.00	2.95	−1.21
70	1.00	1.00	1.00	6.54	−0.66
71	1.00	1.00	1.00	5.22	−1.21
72	1.00	1.00	1.00	6.31	0.07

^a^This experiment was deleted as considered outlier

2 $$ b={\left(X\cdot {X}^T\right)}^{-1}\cdot {X}^T\cdot y $$

Where X^T^ is the transposed of the model matrix.

Taking into account the correlation between the responses, the same conclusions could be obtained by considering the qualitative variable “inspiratory profile” or the quantitative variables “peak of inspiration” or “volume of inspiration”.

The following assumptions were made: the qualitative variable inspiratory profile at six levels has been transformed into a quantitative variable at six levels considering the volume of the inspiration as a new variable; the only two responses were the PC1 and PC2 values of the Scores plot.

Considering that one variable was studied at three levels (*inspiration volume* – X1), two variables were studied at two levels (*type of throat* – X2 and *type of inhaler* – X3), the postulated model was:

3 $$ y={b}_0+{b}_1{X}_1+{b}_2{X}_2+{b}_3{X}_3+{b}_{1-2}{X}_1{X}_2+{b}_{1-3}{X}_1{X}_3+{b}_{2-3}{X}_2{X}_3+{b}_{1-1}{X}_1^2 $$

Where *y* is the PC1 or PC2 value; b_0_ the constant of the multiple linear regression; b_1_ the linear term coefficient of X_1_; b_2_ the linear term coefficient of X_2_; b_3_ the linear term coefficient of X_3_; b_1-2_ the coefficient of interaction X_1_ - X_2_; b_1-3_ the coefficient of interaction X_1_ - X_3_; b_2-3_ the coefficient of interaction X_2_ - X_3_ and b_1-1_ is the quadratic term coefficient of X_1_. The experimental plan is reported in Table II whereas Table IV illustrates the model matrix.

**Fig. 6:**
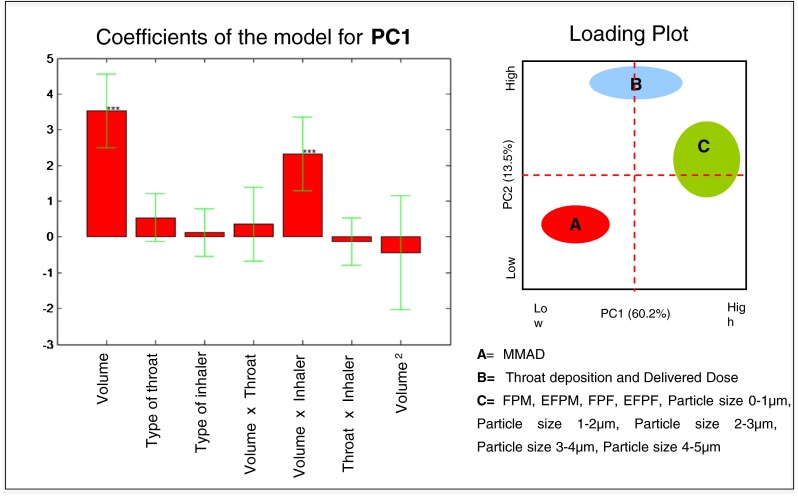
Model coefficients for the PC1 response represented by multiple linear regression. The level of statistical significance is reported according to the convention: * = *p* < 0.05, ** = *p* < 0.01, *** = *p* < 0.001.

**Fig. 7:**
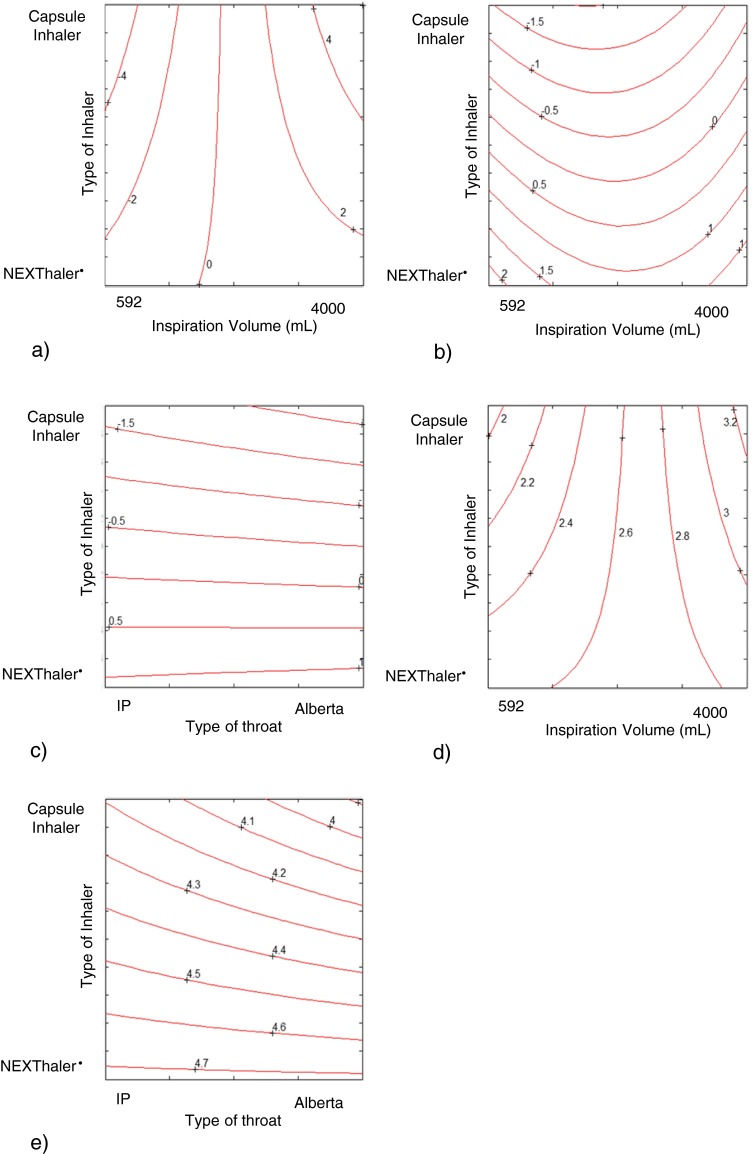
Response surface for the interaction between inspiration volume and type of inhaler on PC1 (7a); type of inhaler and inspiration volume on PC2 (7b); type of inhaler and type of throat on PC2 (7c); type of inhaler and inspiration volume on FF fine particle mass (7d) and type of inhaler and type of throat on FF delivered dose (7e).

The multiple linear regression (MLR) allowed estimating the coefficients of the model for the response PC1 (Fig. 6). The statistically significant coefficients of the model were the linear term of the *inspiration volume* and its interaction with the *type of inhaler*: the higher the volume of inhalation, the higher the value of PC1. High PC1 values means high values of the responses FPM, EFPM, FPF, EFPF, Particle size 0–1 μm, Particle size 1–2 μm, Particle size 2–3 μm, Particle size 3–4 μm, Particle size 4–5 μm and low MMAD value. The *type of throat* had no significant effect on PC1, namely it has no effect on the all the above-mentioned responses. The best way to evaluate the pattern of interaction is to use the response surface graph (Fig. 7).

The response surface shows that PC1 changed more with the variation of the *inspiration volume* when the capsules inhaler was used (Fig. 7a). This means that FPM, EFPM, FPF, EFPF, Particle size 0–1 μm, Particle size 1–2 μm, Particle size 2–3 μm, Particle size 3–4 μm, Particle size 4–5 μm are more influenced by the *inspiration volume* when the capsules inhaler is used.

**Fig. 8:**
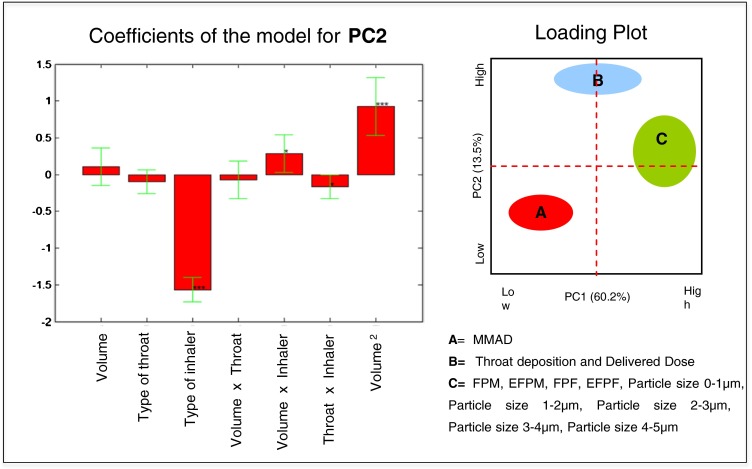
Model coefficients for the PC2 response represented by multiple linear regression. The level of statistical significance is reported according to the convention: * = *p* < 0.05, ** = *p* < 0.01, *** = *p* < 0.001.

In the same way, the multiple linear regression on the matrix of the model allowed estimating the coefficients of the model for the response PC2 (Fig. 8). The statistically significant coefficients of the model were the linear term of the *type of inhaler*, its interaction with the *inspiration volume* and with the *type of throat*, as well as the quadratic term of the *inspiration volume*. The value of PC2, that increases the value of Delivered Dose and throat deposition, decreases by passing from the multidose inhaler to the capsules inhaler (Fig. 8).

Furthermore, the response surface (Fig. 7b) shows how the PC2 changes more with the *inspiration volume* when the capsules inhaler is used, meaning that the throat deposition and Delivered Dose are more influenced by the *inspiration volume* when using the capsules inhaler. This also underscores that the Breath Activated Mechanism, BAM, of the NEXThaler^®^ mitigates the effect of the variability related to the inspiratory capacity by the different asthmatic patients. The minimum flow that ensures the activation of the BAM was lower than the peak of inspiration of all inspiratory profiles investigated.

Analogously, the response surface (Fig. 7c) shows how the PC2 changed more with the *type of throat* when the capsules inhaler is used, indicating that throat deposition and Delivered Dose are more influenced by the *type of throat* when the capsules inhaler is used.

Multiple linear regression on the principal components allows a qualitative global assessment of the influence of the variables on the *in vitro* inhalation performance. In order to obtain a quantitative evaluation, a multiple linear regression on the two main responses described within the PC1 and PC2 (i.e. Fine Particle Mass and Delivered Dose) was performed. As an example, only the MLR on FPM and DD of formoterol fumarate are described below.

**Fig. 9:**
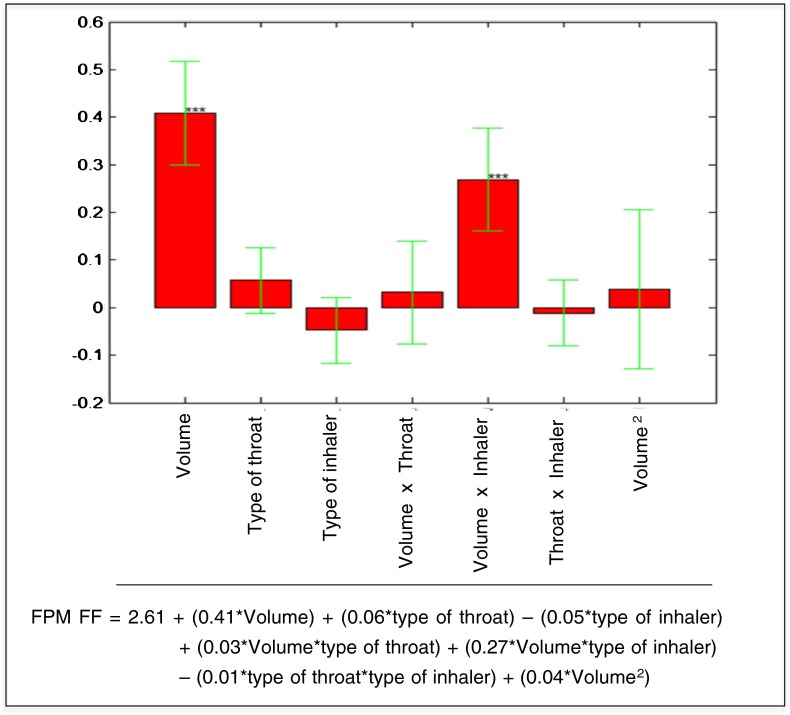
The coefficients of the model and the equation of the model for the formoterol fumarate fine particle mass.

The coefficients of the model and the equation of the model for the formoterol fumarate FPM are shown in Fig. 9. The statistically significant coefficients of model are the linear term of the *inspiration volume* and its interaction with the *type of inhaler*.

The trend of the interactions was evaluated using the response surface shown in Fig. 7d. The FF FPM increases from low to high values of *inspiration volume*: using the NEXThaler^®^ the FPM increased from 2.6 to 2.8 μg moving from low to high inspiration volumes. The FF FPM however varies over a much broader range using the capsules inhaler, from 1.9 to 3.3 μg. This means that the *inspiration volume* has a much greater effect on the FF FPM using the inhaler capsules. The effect of BAM is further confirmed. On the contrary there was no effect of the *type of throat* on the FPM.

The values of the formoterol fumarate FPM for each of the two types of inhaler and with the maximum and minimum inspiration volume were predicted with the model equation and reported in Table V.

**Table V:** Predicted values of Fine Particle Mass (FPM) and Delivered Dose (DD) of Formoterol Fumarate Calculated Using the Model Equation and Different Combination Between Type of Inhaler, Type of Throat and Inspiration Volume, (IP=Induction port; AT=Alberta throat)

Variable combination	
FPM (μg)	
Capsule inhaler + inspiration volume max	3.3
Capsule inhaler + inspiration volume min	1.9
NEXThaler^®^ + inspiration volume max	2.8
NEXThaler^®^ + inspiration volume min	2.6
DD (μg)	
Capsule inhaler + IP	4.2
Capsule inhaler + AT	3.9
NEXThaler^®^ + IP	4.7
NEXThaler^®^ + AT	4.7

**Fig. 10:**
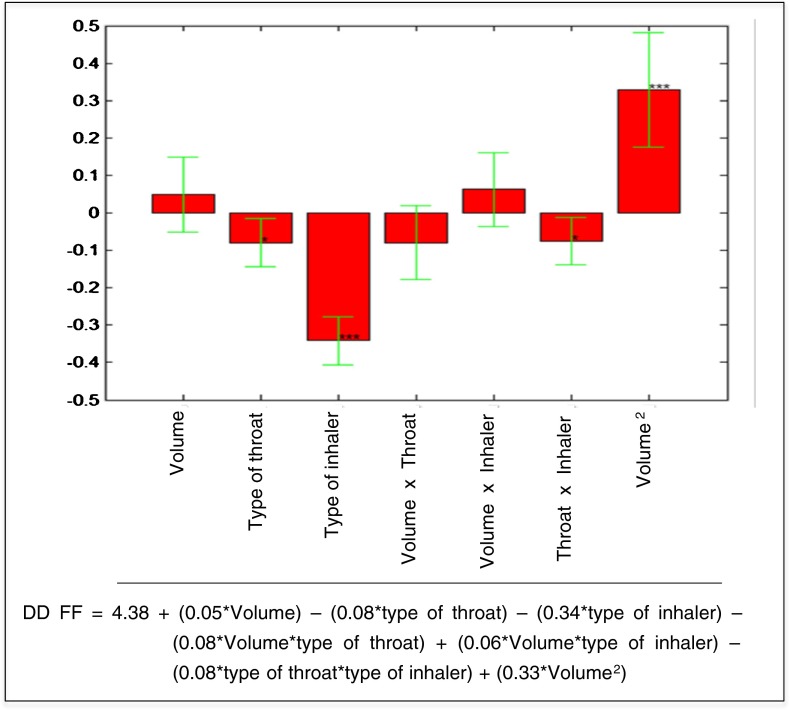
The coefficients of the model and the equation of the model for the formoterol fumarate delivered dose.

The coefficients of the model and the equation of the model for the formoterol fumarate DD are shown in Fig. 10. The statistically significant coefficients of the model were the *type of inhaler*, *the type throat*, the interaction between the *type of throat* and the *type of inhaler*, the quadratic term of the *inspiration volume*.

Moving from the multidose to capsules inhaler the value of the delivered dose decreases. The response surface showed that the multidose released a dose of FF around of 4.7 μg independently from the type of throat employed (Table V and Fig. 7e). When the capsule inhaler was used, the FF DD ranged from 3.9 μg (with Alberta throat) to 4.2 μg (with IP) suggesting that the *type of throat* has a much greater effect on the delivered dose using the capsules device.

## Discussion

The use of DoE and Principal Component Analysis are useful tools in the pharmaceutical development to evaluate the effect of the variables and their interactions on specific answers as well as to improve the quality of the product (21,22). The results here illustrated showed that FF and BDP had a very similar aerodynamic behaviour. This is in agreement with *in vivo* studies where a high amount of the extrafine dry powder fixed combination BDP/formoterol administered via the NEXThaler^®^ was co-deposited in the lungs regardless the pathophysiological condition (23,24).

The DoE responses showed, as expected, an inverse correlation between the values of FPM, EFPM, FPF, EFPF and the value of MMAD since the respirability of a particles is strictly connected to their small aerodynamic size. The type of throat did not influence the responses obtained suggesting that in this case the presence of a laryngeal jet did not affect the impact of drug particles as reported when mono-disperse aerosol particles of DEHS (di-2-ethylhexyl-sebecate) oil with mass median diameters in the range of 2.5–7.5 μm were employed at steady flow rates of 30–90 L/min (14).

Differences were noted between the two types of inhalers: experiments performed with the capsules inhaler showed lower values of DD and throat deposition, compared to those conducted with the multidose inhaler.

The multidose inhaler used is a medium strength inhaler that contains a mechanism activated by the inspiration of the patient (BAM) that avoids the uncontrolled administration of the drug (dose protector). In particular, the device allows releasing the drug only when a specific threshold of inspiration flow rate value is attained (35 L/min) (12,24). Therefore, the observed effect should be attributed to the presence on the BAM that is able to release instantaneously the dose (0.35 s) (12) thus making less relevant the variability linked to the inspiratory capacity of the different asthmatic patients.

Inspiratory profiles generated using a breath simulator had an influence on *in vitro* inhalation performance: FPM and DD obtained using MIN profiles, 10^th^ percentile and 50^th^ percentile were different from the reference condition and the extent of such difference depended on the type of inhaler. In the case of the multidose these differences were not significant, while in the case of the capsule inhaler the FPM and DD values were significantly lower compared to the reference condition.

However, capsule inhalers are a commonly used devices to efficiently deliver several types of medicaments by inhalation (25,26). In particular RS01 was superior in the aerosolization of FF in terms of drug dispersion and emitted dose compared when the same carrier-based formulation was aerosolized using HandiHaler or Turbospin devices (27,28). The motion of the capsule rotating along the minor axis was shown to be the most efficient mechanism in boosting the powder emission. Nevertheless, the different performance between the two inhalers tested in this study has to be attributed to the presence of the BAM in the NEXThaler^®^ which allows to mitigate the effect of the variability linked to the inspiratory capacity by the different asthmatic patients, ensuring the therapeutic efficacy of the drug.

The profiles of patients were described using three parameters: the inspiratory inhalation peak, the duration of inspiration and the volume of inspiration. The peak inhalation and the volume of inspiration were two quantitative variables that demonstrated to represent very well the inspiratory profiles. Thus, it was possible to draw the same results by describing the inspiratory profile in terms of peak inhalation and volume of inspiration. Contrary, the duration of inspiration, had no impact on the experimental responses.

## Conclusions

This work confirms that the approach to drug development required by the guideline ICH Q8 “Pharmaceutical Development” allows identifying and explaining in a systematic and robust approach the effect of the variables on the critical quality attributes of new medicinal product.

The outcome of experimental design gave the possibility to identify the most significant variables affecting the results and to build model equations allowing the prediction of the experimental results in the domain.

The multivariate statistical approach allowed highlighting the major novelty of this work, namely the demonstration that the multidose inhaler equipped with the BAM mechanism, , differently from the single dose capsule inhaler, released efficiently a full therapeutic dose with a high fine particle dose independently from the flow rate applied.

The reproduction of actual inspiratory profiles of asthmatic patients, by means of a breath simulator, was a useful tool to mimic *in vitro* the *in vivo* conditions.

Finally, the type of throat (induction port or Alberta throat) coupled with the impactor did not affect the aerodynamic particle size distribution of the investigated drugs, this represents a further significant new contribution of the present work.

## Abbreviations

AT: Alberta throat
BAM: Breath activated mechanism
BDP: Beclomethasone dipropionate
CQAs: Critical quality attributes
DD: Delivered dose
DPI: Dry powder inhaler
EFPF: Extra fine particle fraction
EFPM: Extra fine particle mass
FF: Formoterol fumarate
FPF: Fine particle fraction
FPM: Fine particle mass
IP: Induction port
MLR: Multiple linear regression
MMAD: Mass median aerodynamic diameter
NGI: Next generation impactor
PCA: Principal components analysis
PIF: Peak inspiratory flow
QC: Quality control
USP: United States pharmacopeia
